# Modeling a Standard Loop-Mediated Isothermal Amplification Reaction and Its Modification Involving Additional Inner Primers

**DOI:** 10.3390/biom15050690

**Published:** 2025-05-09

**Authors:** Liana U. Akhmetzianova, Constantin I. Mikhaylenko, Dmitry A. Chemeris, Valery D. Khairitdinov, Assol R. Sakhabutdinova, Irek M. Gubaydullin, Ravil R. Garafutdinov, Alexey V. Chemeris

**Affiliations:** 1 Institute of Biochemistry and Genetics, Ufa Federal Research Center, Russian Academy of Sciences, 71, prosp. Oktyabrya, Ufa 450054, Russia; www.lianab@mail.ru (L.U.A.);; 2 Department of Technology, Ufa State Petroleum Technological University, 1, st. Cosmonauts, Ufa 450064, Russia; yeevalera@gmail.com (V.D.K.); irekmars@mail.ru (I.M.G.); 3 Mavlyutov Institute of Mechanics, Ufa Federal Research Center, Russian Academy of Sciences, 71, prosp. Oktyabrya, Ufa 450054, Russia; 4 GENVED LLC, 25/1, Parshina st., Moscow 123103, Russia; 5 Institute of Petrochemistry and Catalysis, Ufa Federal Research Center, Russian Academy of Sciences, 141, prosp. Oktyabrya, Ufa 450075, Russia

**Keywords:** loop-mediated isothermal amplification (LAMP), LAMP nomenclature, lamplicons, pseudo-hemi-nested LAMP (phn-LAMP), primer design

## Abstract

Loop-mediated isothermal amplification (LAMP) was developed a quarter of a century ago, but it is still not exactly clear how this reaction proceeds. Only a few articles have focused on the kinetics of LAMP and the types of products formed. In this work, 10 types were identified and named. A basic dumbbell structure, Z6_dmb(1), consists of six zones and triggers the LAMP cycle. Due to self-priming, Z6_dmb(1) transforms into hairpin structure Z9_hp(1) and then into linearized strand Z9_li(1), carrying also strand Z6_dmb(2). Through similar transformations, it again generates strand Z6_dmb(1), completing the first LAMP cycle and starting a new one. The next stage of the exponential phase starts from two Z15_hp hairpin structures generated in the LAMP cycle, which next turn into Z15_li → Z27_hp → Z27_li → Z51_hp → and so forth. Modeling of a new type of the reaction, namely, pseudo-hemi-nested LAMP (phn-LAMP), was carried out. phn-LAMP involves three inner primers: two forward (FIP and extraFIP) and one backward inner primer, or vice versa. phn-LAMP has an advantage over LAMP involving loop or stem primers and over MIP-LAMP (multiple inner primers).

## 1. Introduction

After PCR, the second most widely used reaction for amplification of specific fragments of nucleic acids with exponential accumulation of products is loop-mediated isothermal amplification (LAMP) [1]. The popularity of this method, which is rather complex in terms of primer system design, is explained by the feasibility of its application without expensive equipment and in point-of-care (POC) assays, which allow for quickly obtaining the necessary diagnostic information. The LAMP method was actively developed during the COVID-19 pandemic, since it provided rapid and reliable detection of the SARS-CoV-2 coronavirus [2,3,4].

**Figure 1:**
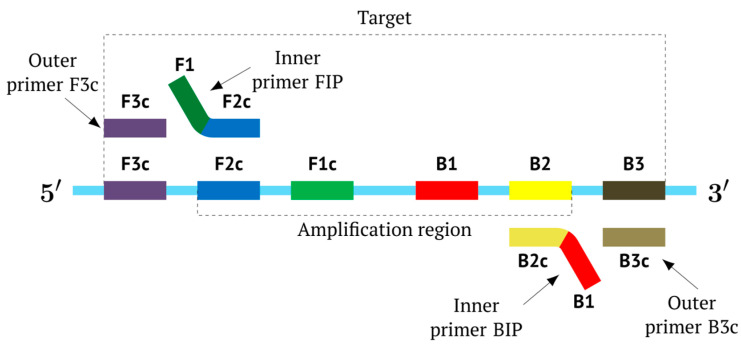
Disposition of primer annealing zones in standard LAMP reaction and their conventional designations.

In the original paper [1], two outer primers—F3 (forward) and B3 (backward)—and two inner primers—FIP (forward inner primer) and BIP (backward inner primer)—were proposed for the LAMP reaction. FIP and BIP each carry a pair of annealing sites (F1c|F2 and B1c|B2, respectively) present on the target. The locations of these are presented in Figure 1, which shows a standard scheme of LAMP.

Later, to accelerate the collection of results, the authors of the LAMP reaction proposed a modification, which involves six primers specific to eight target sites [5]. Loop primer F and loop primer B were added, which are annealed on target sites between zones F2c and F1c and between B1 and B2, respectively. Then, other authors proposed a type of LAMP with stem primers whose annealing sites are located between primers F1c and B1 [6]. Later, the use of multiple inner primers FIP and BIP was proposed for the LAMP reaction, for the same purpose of accelerating the obtaining of results; accordingly, this approach is called MIP-LAMP (multiple inner primers), and together with loop primers and outer primers, the number of annealing sites for them on the target reached 14 in that work [7]. On the one hand, the introduction of additional loop and stem primers accelerates the amplification; on the other hand, the length of the target required for detection increases, which may be problematic in some cases, when, for example, old samples are analyzed. In addition, due to the larger number of primers, the probability of dimer formation increases, consequently leading to false positives.

**Figure 2:**
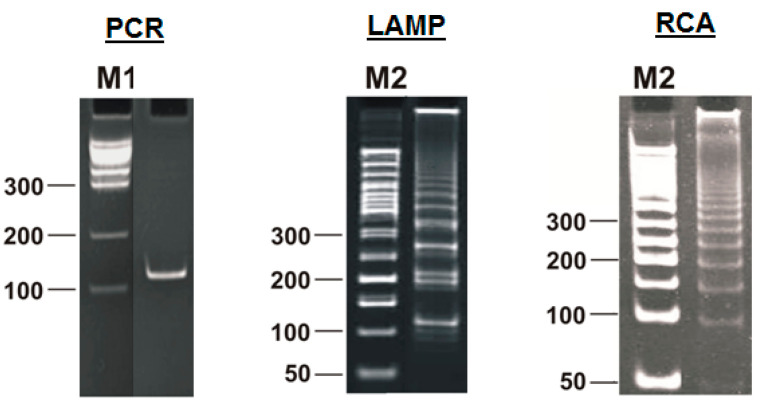
Products of different amplification reactions: amplicon from polymerase chain reaction (PCR), lamplicons from loop-mediated isothermal amplification (LAMP), and concatemers from rolling circle amplification (RCA). M1 and M2: standard marker ladders.

Due to the increased number of primers and their special design, even the standard LAMP reaction is much more complicated than classic PCR; this conclusion, among others, can be reached by looking at the animation from Eiken Chemical Co. (https://loopamp.eiken.co.jp/en/lamp/0211.html (accessed on 8 May 2025)) and the video from New England Biolabs (https://www.neb.com/en/tools-and-resources/video-library/loop-mediated-isothermal-amplification-lamp-tutorial (accessed on 8 May 2025)). That conclusion is also confirmed by the results of gel electrophoresis of the products of this reaction: a characteristic ladder of DNA bands of increasing size, in contrast to conventional PCR (Figure 2).

Similar ladders of DNA bands—in the form of increasing products of amplification reactions—are also produced by other methods of nucleic-acid amplification, but they have a different inner structure. Due to the fundamental differences between amplicons of PCR and the products generated during LAMP, different groups of authors [8,9,10,11] have introduced the name “lamplicon” or “LAMPlicon” for the latter, which we will use here.

A quarter of a century has passed since the invention of LAMP [12], and many studies have been conducted with the use of LAMP reactions, including the development of new types of LAMP and numerous approaches for the detection of the results (see reviews [13,14]). Nevertheless, there is no detailed description of all possible types of lamplicons and their composition, except for a few papers. For instance, only a decade and a half after this method was developed, an article was published in which researchers developed a mathematical model of this reaction, noting that to their knowledge, this was the first attempt to quantitatively model LAMP [15]. At the same time, those authors limited themselves to constructing an S-shaped Richard curve and estimating the multiplication of lamplicons in time, without going into their types. The next similar work appeared only 5 years later [16]. Then, in 2020, a stoichiometric and pseudo-kinetic model was constructed, contributing to a better understanding of the LAMP reaction by classifying its products [17]. In 2022, a paper was published about the exponential phase of the LAMP reaction and was focused on concentrations of the different components of the reaction, thus providing a number of recommendations to practitioners [18]. At the same time, those authors noted that this topic only recently attracted the attention of scientists and stated that theoretical elucidation of LAMP will contribute to the optimization of experiments, by reducing the time needed to select the right parameters. Finally, th kinetics of elementary steps of LAMP were recently studied, and the speed of Bst polymerase when passing through different regions of templates was evaluated, showing that the speed decreases markedly when this enzyme displaces the DNA strand [19].

Nonetheless, the LAMP reaction deserves more attention from the point of view of studies on the formation of different types of lamplicons, and the present article is devoted, among other things, to obtaining this missing information. A new rather promising type of LAMP reaction is also described here: pseudo-hemi-nested LAMP (phn-LAMP).

## 2. Results and Discussion

### 2.1. Nomenclature of Target Zones in the LAMP Reaction

**Figure 3:**
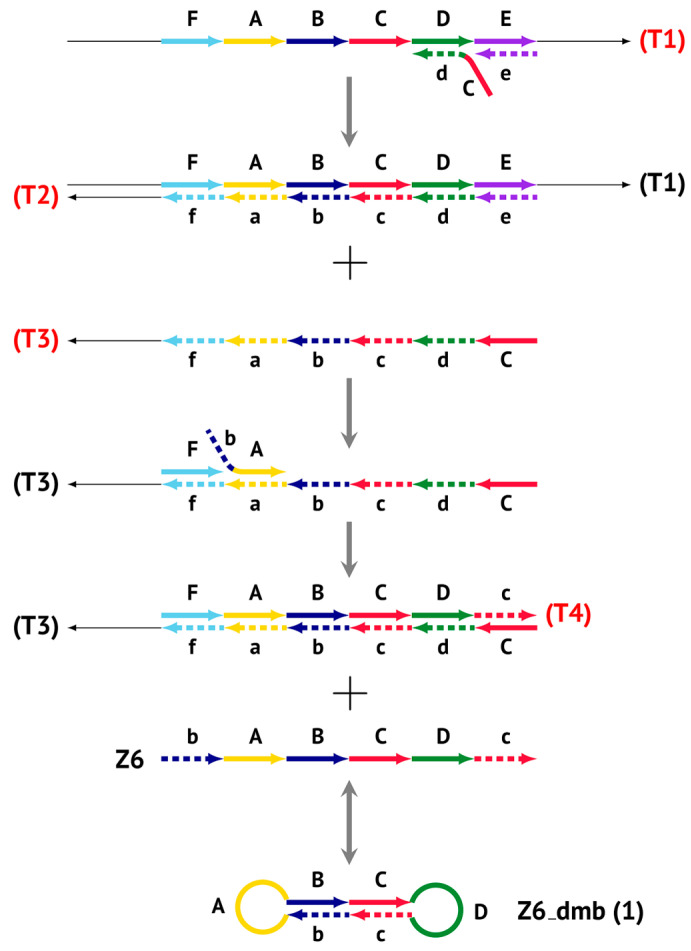
Initial steps of the LAMP reaction (explanation in text).

As noted above, the structure of lamplicons in the LAMP reaction is extremely complex, and their diversity cannot be described simply. The first obstacle is the nomenclature of zones in a target, which was proposed in 2000 (Figure 1). According to this, the zones on one DNA strand are designated both with and without the letter ‘c’ (meaning ‘complementary’). This approach cannot be considered highly correct. To make it easier to operate with primers and lamplicons, the names of primer annealing zones should be modified. We propose single-letter symbols for all primer annealing zones. Instead of zones F3c, F2c, F1c, B1, B2, and B3, the following should be used: F, A, B, C, D, and E and, respectively f, a, b, c, d, and e (Figure 3). The annealing zones of loop primers LF and LB can be labeled, for example, as X, Y and x, y. In general, all these zones can be labeled as Z and z (meaning a zone), supplied with a number corresponding to an ID number of such zones in a particular lamplicon. The primers used in the LAMP reaction will have the following letter designations, either uppercase or lowercase (reflecting their annealing on the starting or complementary strands): outer primer F3c will be “F”, the other outer primer B3 will then appear as “e”, while loop primers LF and LBc will be “X” and “y”, respectively. As for composite primers FIP and BIP, given that they carry two annealing zones (F1c|F2 and B1c|B2), they can each be labeled with two letters: 5′-bA-3′ and 5′-Cd-3′, respectively. Furthermore, where a direction of a zone sequence is not specifically indicated (including for primers), it will be 5′→3′ by default. The distinct feature of our proposed nomenclature is that all zones belong to one strand, and the letter ‘c’ is no longer required. This is because on a presumed upper strand, letters designating the zones are capitalized, whereas on the complementary (lower) strand (not shown in Figure 1), they are lowercase.

Nevertheless, we by no means propose replacing the already customary names of primer bands in LAMP reactions, as will become evident in the description below. The replacement by a one-letter designation has only a technical purpose and is necessary only for a better understanding of processes taking place in this very complicated reaction. On the other hand, it is necessary to recognize that many additional designations have to be introduced, without which it is impossible to paint a complete picture of the processes occurring during LAMP, but these names do not detract from the general logic of this reaction.

Thus, all zones on the initial DNA strand serving as a target (T1) or templates T2, T3, and T4 for primer annealing in a LAMP reaction without loop primers can be depicted as 5′-FABCDE-3′, where dotted lines symbolize flanking sequences of indefinite length. When a LAMP reaction is carried out with loop primers (which are outside the scope of this paper), zones in the T1 target may be labeled 5′-FAXBCYDE-3′.

It should be noted that the structures described below may occur with certain probabilities during LAMP, in accordance with the competition between primer annealing and self-priming, which in turn depends on specific nucleotide sequences of the various zones in the target. Because they may differ greatly among different objects under study (targets), we will have to consider here all possible types of formation of hairpin structures and annealing zones both for primers and for 3′ ends of the generated lamplicons, which provide self-priming.

### 2.2. The Preamplification Phase of the LAMP Reaction

The main prerequisite for a LAMP reaction is early annealing of inner primers at the initial stage of the preamplification phase, so that the strands built are displaced and become single-stranded (Figure 3). Then, they become suitable for the annealing of new primers. This occurs due to delayed annealing and elongation of outer primers, which is ensured by the presence of the latter at a lower concentration in the reaction mixture.

The processes depicted above can be represented more compactly using the single-letter symbols assigned to the various zones. Such a description is also important because it indicates that, in spite of the same size, different lamplicons have different compositions of zones; this state of affairs will be especially noticeable in the transition of this reaction to the exponential phase. For example, annealing of primers Cd and e sequentially on the T1 target will give rise to the following structures: 
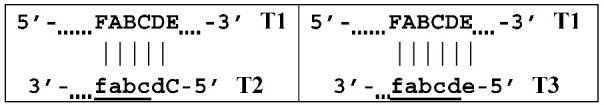
 where vertical lines collectively symbolize hydrogen bonds between complementary nucleotides in specific zones, and underlining indicates new strands being built from the primer, which itself remains underlined. The primer zones that served for self-priming will not be underlined either. Such strands together with the primer, being a whole, serve as new templates and are presented without underlining. Alphanumeric names denote the type of DNA strand built from the annealed primer. A similar coding will be used to describe processes occurring in the exponential phase of LAMP, including the main LAMP cycle, as well as the phn-LAMP reaction.

Because the T2 strand will be displaced during the construction of the T3 strand after elongation of outer primer e by displacing the activity of polymerase, the template becomes single-stranded and suitable for subsequent annealing of the following primers on it: bA and F. 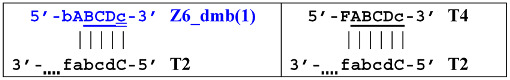


Elongation of primer bA will form the **bABCD****c** Z6_dmb(1) structure, where the double underlining indicates the zone mediating self-priming. By elongating the F primer, however, it will displace the Z6_dmb(1) strand, which is the first dumbbell-like structure. This is the most important outcome of the preamplification phase of the LAMP reaction.

### 2.3. Types of Products in the LAMP Reaction

The first dumbbell-like structure 5′-**bA****BCDc**-3′ Z6_dmb(1) generated during the preamplification phase is one of basic elements of any LAMP reaction and starts the cascade of subsequent events. Nevertheless, before proceeding to a detailed review of the processes within the LAMP cycle as well as outside it, it is necessary to focus on different types of lamplicons arising during LAMP. Namely, previously, Kaur et al. [17] have identified four categories among LAMP reaction products: SL (single loop), T (terminated), SS (single-stranded), and PDS (partially double-stranded), and affixed Roman numerals to some of them: I, II, and III. In another article [18], investigators analyzed the second phase of the LAMP reaction starting from dumbbell-like structures, calling it IDEA (isothermal dumbbell exponential amplification), using slightly different starting structures, which were designated as a short dumbbell, middle dumbbell, and long dumbbell; all differences among them consisted of different lengths of small additional nucleotide sequences in the region between zones F1c and B1, according to Figure 1. Self-priming of the initial dumbbell results in the formation of hairpin B2c = Hp1b. After several displacements of strands, a hairpin emerges that is symmetric to it; this hairpin was named hairpin 1 F2c = Hp1f.

Nonetheless, some other types of lamplicons also arise during the LAMP reaction, which also need to be examined (Table 1).

**Table 1:** Types of products in classical loop isothermal amplification—LAMP.

Picture	Name	Brief Description
LAMP reaction intermediates		
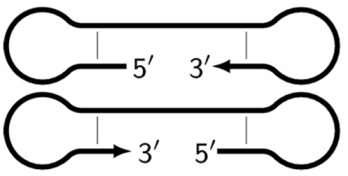	Z_dmb(1)Z_dmb(2)	Dumbbell-like structures in the form of single- or double-stranded DNA. The letter Z (zone) is accompanied by a number corresponding to the ID number of zones in specific products. Z6_dmb(1) and Z6_dmb(2) are the main types of lamplicons that trigger cascades of subsequent processes of enzymatic construction of DNA strands and ensure exponential amplification. In fact, strands bABCDc and CdcbaB are dual mirror twins with respect to each other.
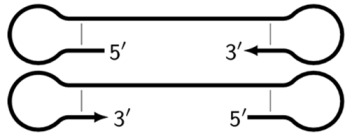	Z9_dmb(1)Z9_dmb(2)	Elongated dmb structures in the form of single/double-stranded DNA similar to Z6_dmb but differing in the length owing to a different number of zones, e.g., Z9_dmb or Z15_dmb.
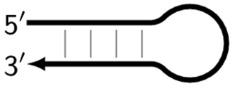	Z_hp	Hairpin-like structure of single-stranded DNA (loop) and double-stranded DNA (stem). Arises from Z_dmb, e.g., Z6_dmb gives rise to Z9_hp.
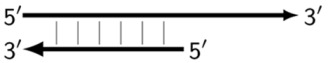	Z_li	Single-/double-stranded DNA structure (linear) resulting from linearization of hairpin structure Z_hp and then evolving into a new, larger Z_hp structure and also generating a smaller Z_dmb structure(s).
Terminated (stop) lamplicons in LAMP reaction		
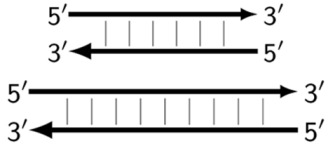	ZzS	Double-stranded DNA structures generating by polymerase from structures like Z_li or Z_dmb (here, generated from Z6_dmb, Z9_li or Z9_dmb, respectively) and not participating in further amplification.
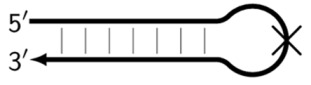	Z_hpS	Hairpin-like structure in the form of double-stranded DNA (stem) and single-stranded DNA (loop), on which (on whose loop) the next primer cannot anneal, as indicated by the crossing out of the loop.
Other unstable products of LAMP reaction		
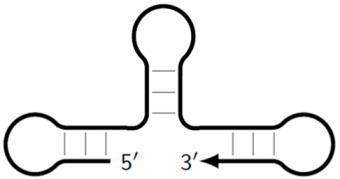	Z_dmb_hp	Dumbbell-like structure with looping in the stem part, on whose loop one of the inner primers is also able to anneal, followed by elongation and strand displacement.
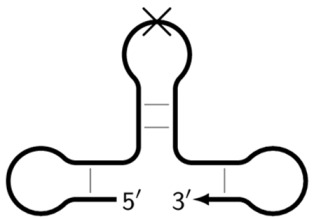	Z_dmb_hpS	Dumbbell-like structure with a hollowing out in the stem part, on whose loop the inner primers are unable to anneal, as indicated by the crossing out of the loop.
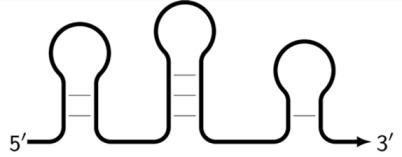	Z_mtlp	Late-onset single-/double-stranded DNA containing multiple different loops that can also participate in further amplification.
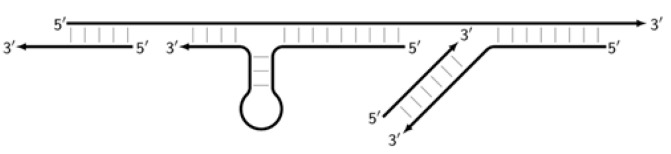	cauliflower-like	High-molecular-weight single-/double-stranded DNA arising at late stages, carrying strands annealed on each other in a chaotic manner. Limitedly suitable for further amplification.

The probability of formation of certain types of products depends on their GC content and on stacking interactions of neighboring nucleotides in specific zones and cannot be predicted.

It should also be pointed out that in the types of LAMP reaction involving loop primers and stem primers, other types of products appear as semi-dumbbell (sdmb) structures, but we will not touch upon them here. Among the final products of the LAMP reaction are DNA molecules with double-stranded regions, representing the target with flanking single-stranded regions (shown by dotted lines) of unknown length: 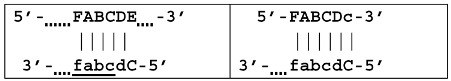


The number of such molecules will approximately correspond to the initial number of copies of the desired sequence, and they will not make a serious contribution to the total amount of DNA produced during the LAMP reaction.

### 2.4. The Basic LAMP Cycle

**Figure 4:**
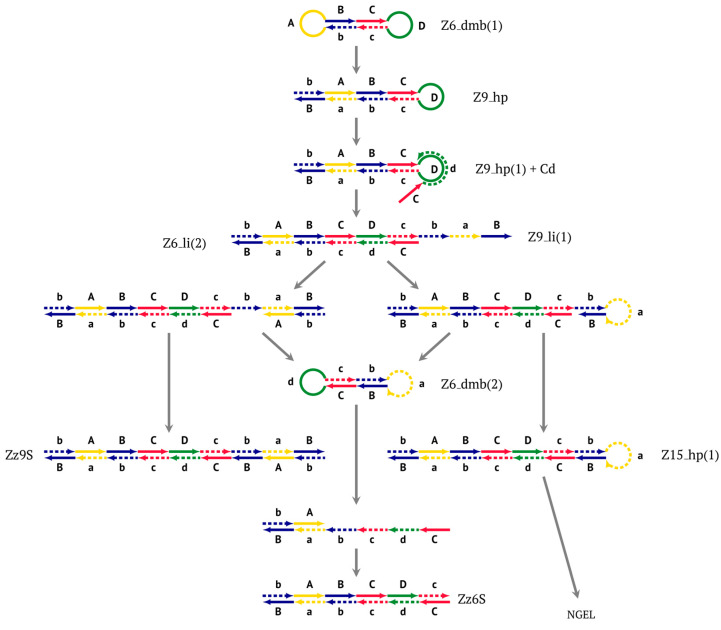
The scheme of events at the first stage of the exponential phase of the LAMP reaction.

After the appearance of the first dumbbell-like structure Z6_dmb(1), exponential amplification begins as the LAMP cycle, which continues by releasing a number of products into the outer contour, in which the next generation of extended lamplicons (NGEL) takes place and similar dumbbell-like Z6_dmb structures are formed, initiating similar LAMP cycles. The processes taking place are summarized in Figure 4.

**Figure 5:**
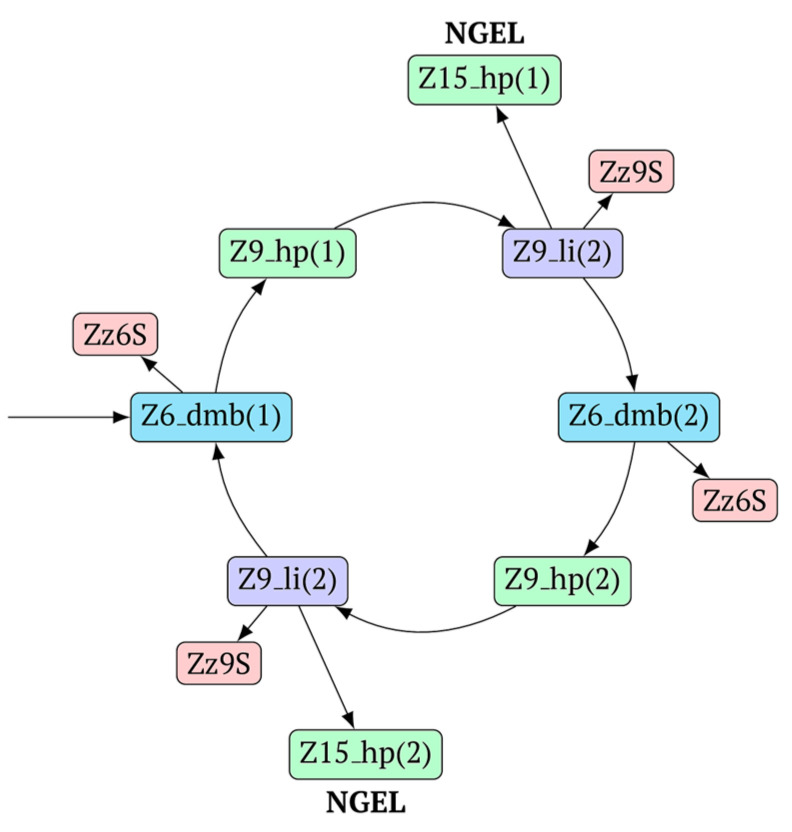
The main (first) LAMP cycle of the exponential phase in the classic version of LAMP, beginning with the **Z6_dmb(1)** loop of the structure emerging during the preamplification phase. Terminated lamplicons are red. Z15_hp lamplicons enter the outer contour where NGEL takes place (other explanations are in the text).

The most interesting is the LAMP cycle, which repeats many times and occurs at the next stages in the course of the formation of larger lamplicons during NGEL when they return to the main lamplicon Z6_dmb. The sequence of events in the LAMP cycle is depicted in Figure 5.

Upon entering the LAMP cycle, the **bABCD****c** strand appears suitable for annealing with the Cd primer, leading to the terminated Zz6S lamplicon (shown hereafter in red), the amount of which will increase during the LAMP reaction: 
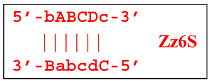


Moreover, only Zz6S and similar terminated double-strand lamplicons ZZS and also Z_hpS lamplicons are visible when products of the LAMP reaction are separated by gel electrophoresis. As for some of the following single-/double-stranded lamplicons, due to the isothermal nature of the process, at every moment among many similar structures there will be those differing from each other by an unequal number of nucleotides. Therefore, this situation will interfere with the formation of discrete DNA bands of equal or similar sizes visible to the naked eye in the gel. It is also worth noting that the probability of formation of the above-mentioned terminated double-stranded Zz6S lamplicon is rather high, if we take into account that the Cd primer will be annealed on the bABCDc structure in two zones (italicized here) at once, thus representing serious competition to self-priming, which means annealing only by a single zone.

Nevertheless, the dumbbell-like structure of **bABCD****c**—owing to self-priming (annealing of zone ‘**c**’ on zone ‘C’) and subsequent strand elongation—gives rise to a new hairpin structure (Z9_hp_D, where hp is a hairpin) with a loop in the form of zone D: 
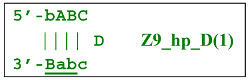


At the same time, primer Cd can be annealing (at first with one zone d) on the single-stranded loop formed by the D zone, whose elongation by DNA polymerase will cause the displacement of the strand and will transform the Z9_hp_D strand into a linearized version [Z9_li (linear)], which will have a double-stranded section carrying the Z6_dmb(2) strand: 
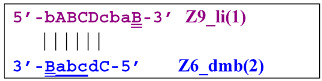


On the Z9_li(1) strand, the bA primer is also able to anneal (with two zones), leading to both a terminated lamplicon 
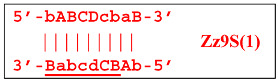
 and to the displacement of the **Cdcba****B** strand, forming in turn the second main dumbbell-like structure Z6_dmb(2) with a symmetric sequence in relation to Z6_dmb(1). They are in fact dual mirror twins. Indeed, the main dumbbell-like lamplicons in the form of strands Z6_dmb(1) and Z6_dmb(2) are fully complementary; if they are present in the reaction mixture in large amounts at late stages of the LAMP reaction, then they can form a double-stranded terminated structure Zz6S by simply annealing on each other. Dumbbell-like structure Z6_dmb(2) is able to elongate itself by means of polymerase to form a new hairpin with a loop in the form of the “a” zone: 
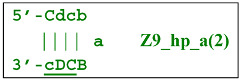


Annealing of primer bA (initially with one zone A) on the a loop of the Z9_hp_a strand and its elongation will enable the annealing of primer Cd (with two zones) and the formation of a number of structures, among which is terminated lamplicon Zz9S(2), which has a sequence different from the similar previous Zz9S(1) lamplicon: 
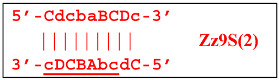


The **bABCD****c** strand (built from primer bA)—displaced by polymerase as a result of primer Cd elongation—will repeat the structure of the first base lamplicon Z6_dmb(1), which will start a new cycle: 
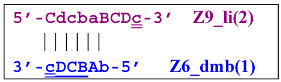


Thus, within one LAMP cycle in the classic version of this reaction, six intermediate products and four terminated lamplicons will be generated as well as two elongated hairpin structures Z15_hp, from which other structures of both larger and smaller sizes will arise later. Here, we can also note that both within the LAMP cycle and during the subsequent multiplication processes, only “c” and “B” act as self-priming zones. This phenomenon should be taken into account when primer systems are designed, with special attention to the 5′ ends of primers FIP (bA) and BIP (Cd) because the 3′ ends of their complementary strands will be elongated by polymerase during self-priming.

### 2.5. Continuation of the LAMP Reaction Beyond the Basic LAMP Cycle

Thus, two different Z15_hp strands, once outside the LAMP loop, will lead to further growth of DNA strands during the LAMP reaction and form new self-extending hairpin structures; because they are symmetric, it is enough to trace descendants of only one: 
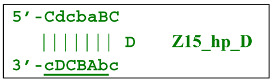


The Cd primer will anneal on the D loop formed in Z15_hp (initially with one zone d) followed by linearization of the strand, resulting in a new single/double-stranded structure: 
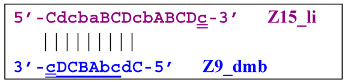


On the remaining single-stranded site, another primer, Cd, will be annealed, causing the previous CdcbABCDc (Z9_dmb) strand to be displaced and causing the formation of a terminated double-stranded lamplicon, which already consists of 15 zones: 
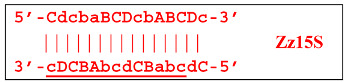


As for the fate of the CdcbABCDc strand (Z9_dmb), it may become a Z15_hp lamplicon or form another lamplicon as a Z_hpS hairpin structure owing to self-annealing: 
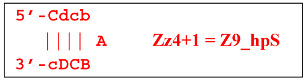


This Z9_hpS lamplicon is a terminated structure because it is a hairpin on which none of the primers present in the reaction mixture can be annealed. This and similar structures will move disproportionately to the location corresponding to the total length of the nucleotide sequence during gel electrophoresis owing to the presence of a single-stranded region with different electrophoretic mobility. Thus, as stated above, during the LAMP reaction, terminated lamplicons are formed, representing not only double-stranded DNA fragments but also some hairpin structures unsuitable for primer annealing on them. This notion also explains the presence in the electrophoretic image of binary and even triple DNA bands with similar sizes and characterized by slightly different mobility in the gel.

When hairpin structure Z15_hp_D is generated, another self-priming of the created structure will occur with intermediate linearization and subsequent formation of a new hairpin: 
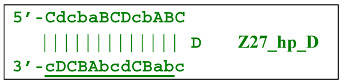


Upon annealing of another Cd primer on the Z27_hp_D hairpin structure (initially with a single d zone), hairpin opening and subsequent linearization of the strand will again occur: 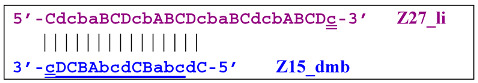


Two more primers, bA and Cd, can anneal on the Z27_li(2) strand, respectively, leading to this structure: 
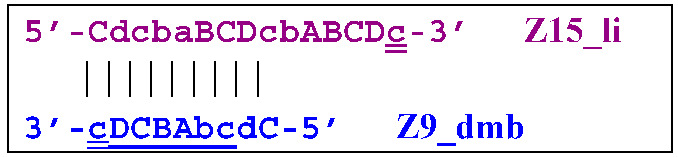
 and in the latter case with the Cd primer, to a terminated lamplicon with 27 zones: 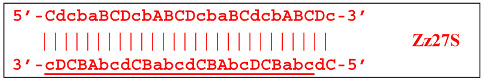 and also to displaced strands Z15_dmb and Z18_dmb.

At the same time, for the displaced strands Z15_dmb and Z18_dmb, there are already three types of self-forming hairpin structures, with subsequent completion of some of them. Thus, the following conversions will occur with the Z15_dmb strand: 
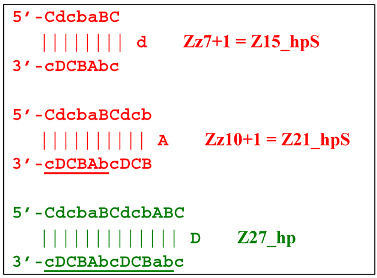


If the first two lamplicons, Z15_hpS and Z21_hpS, are terminated, then lamplicon Z27_hp will turn into structure Z27_li owing to annealing of primer Cd on it (at first by one zone d), which will then become structure Z51_hp and start a new NGEL stage of the LAMP reaction.

The transformation of the Z15_dmb strand will not finish here: two primers, Cd and bA, will be able to anneal on it, and if the former forms a terminated Zz15S lamplicon, the latter will give rise to another dumbbell-like structure bABCDc Z6_dmb(1), which will serve as an entry structure into a new LAMP cycle: 
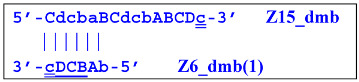


As for lamplicon Z18_dmb’s behavior, it will be similar to that of the Z15_dmb lamplicon, but with some differences. Lamplicon Z18_dmb will also form three hairpin structures: 
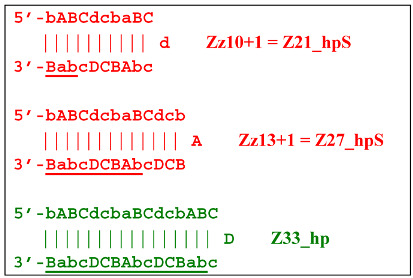


If the first two structures, Z21_hpS and Z27_hpS, are terminated, the last Z33_hp will continue the multiplication of DNA strands, entering a new NGEL stage of the LAMP reaction, and will continue to develop lamplicons of its size series. It can be observed here that the Z21_hpS structures that formed from strands Z15_dmb and Z18_dmb, while having the same size, have different compositions, including different loops, A and d, respectively.

**Figure 6:**
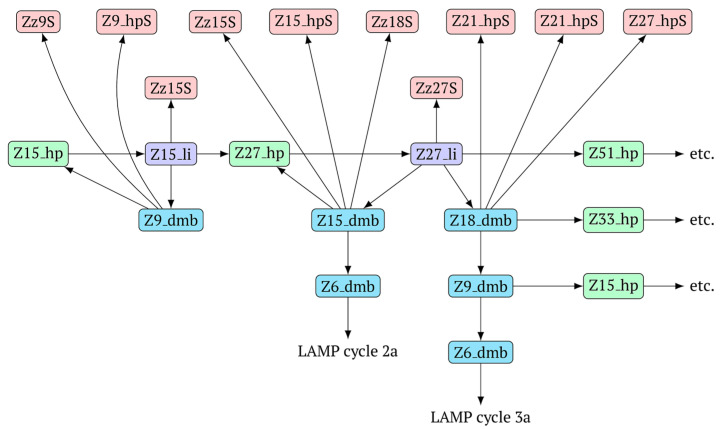
The scheme of the LAMP reaction outside the LAMP cycle after the release of Z15_hp during NGEL into the outer contour.

In addition to all kinds of hairpin structures arising due to self-annealing, primers bA and Cd can be annealed on the Z18_dmb strand. In this case, elongation of the latter primer will lead to a terminated Zz18S lamplicon. In contrast, elongation of primer bA will lead to a dumbbell-like Z9_dmb lamplicon and through it both to the Z15_hp strand in the direction of increasing product length and to the Z6_dmb(2) lamplicon in the direction of decreasing length, thereby starting its LAMP cycle: 
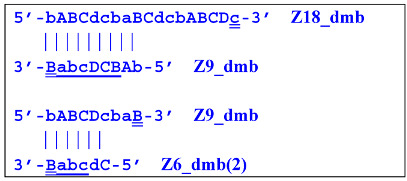


The above-mentioned amplification processes in the LAMP reaction for one lamplicon Z15_hp(1) formed in the first LAMP cycle can also be displayed as a scheme. This scheme implies that for a second (similar) lamplicon Z15_hp(2) formed in the same LAMP cycle 1, the outcomes will be symmetric, with the only difference being that the loop zones will be different (Figure 6).

As evident from the scheme in Figure 6, after the formation of lamplicon Z27_li and the displacement of strands Z15_dmb and Z18_dmb, two new main dumbbell-like structures Z6_dmb are formed, which will start new LAMP cycles. Considering that the reactions are symmetric, in the scheme, they are marked 2a and 3a, implying that there will also be similar LAMP cycles 2b and 3b during this exponential NGEL phase.

Regarding the growth of strands during the LAMP reaction, owing to self-priming, the increase in the number of zones in the lamplicons obeys the following formula:

**Z_i_ = 2Z*****_i__−__1_*****− 3**

Thus, the main pathway of generation of lamplicons in the direction of their elongation looks as follows: Z6 → Z9 → Z15 → Z27 → Z51 → Z99 and so forth. For the other pathway, the following series is lined up: Z18 → Z33 → Z63 → Z123 and so forth. In addition to them—because there are a lot of places for primer annealing in lamplicons of increased size, including on the displaced strands—a variety of lamplicons can be formed whose lengths are also multiples of three. Terminated lamplicons will also have a number of zones that is a multiple of three. Nonetheless, it should be kept in mind that not all possible lamplicons will be produced in appreciable quantities, and whether they will be produced at all depends on specific features of nucleotide sequences of these or other zones in the selected target.

**Figure 7:**
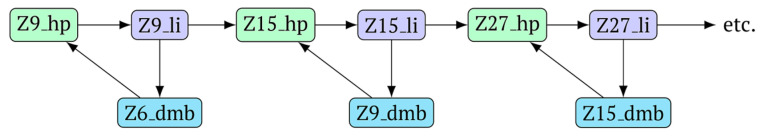
Cycling of some lamplicons in the LAMP reaction during the LAMP cycle and NGEL.

In general, during the LAMP reaction in its exponential phase, the following cyclic processes occur in the main size series, as shown in Figure 7. At the same time, for each of these types (Z_dmb, Z_hp, and Z_li), the composition of the zones will slightly change, but their number within specific dimensional boundaries will be unchanged.

As one can see in Figure 6 and Figure 7, Z_dmb, Z_hp, and Z_li types of lamplicons are capable of transforming into each other, while generating many other lamplicons of different types.

Furthermore, during the exponential phase of the LAMP reaction up to its plateau, various other variants with different sizes of lamplicons can be formed owing to possible hairpin looping due to regularly repeating elements in the form of different Z_mtlp structures, and also due to the formation of other unpredictable structures. This is because in the reaction mixture after some time, a huge number of single-stranded DNA molecules emerge over large regions complementary to each other; these are called “cauliflower-like” structures. At the same time, all these diverse DNA molecules contribute to the overall DNA amplification when they are detected by means of nonspecific fluorescence with intercalating dyes such as SYBR Green I or with the help of byproducts of the DNA polymerization reaction. In these cases, turns of the DNA helix or consumed dNTPs, which transformed into dNMPs and pyrophosphates with a proton release, “work” for detection.

### 2.6. Pseudo-Hemi-Nested LAMP(phn-LAMP) Reaction

Of separate interest are LAMP reactions with an accelerated type of amplification due to additional primers. Neither loop nor stem primers give a very significant increment, and LAMP reactions with multiple inner primers, some which have been proposed earlier [7,20,21,22], look more promising. Nonetheless, apparently due to a significant increase in target length and elevated probability of primer dimerization, these types of LAMP reactions have not become popular. In particular, during the pandemic of novel coronavirus SARS-CoV-2, one paper described the RT-MIPLAMP method. When two pairs of inner primers were used, this method showed an advantage in sensitivity and speed of obtaining results as compared to the conventional RT-LAMP reaction; however, when the number of inner primers was increased to three pairs, nonspecific amplification began [22]. Also, a serious disadvantage of such LAMP reactions with an increased number of inner primers is the greater length of the target.

**Figure 8:**
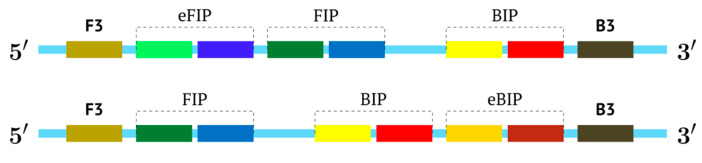
Schemes of primer annealing zones on a target in a phn-LAMP reaction of two types, and their usual names with additional primers, eFIP and eBIP, where “e” means “extra”.

An intermediate option (a compromise) could be the use of only one additional inner primer, and this approach would resemble semi-nested PCR. In the process, however, nested primers enter amplification later, which arises due to temperature differences in primer annealing or by some barrier method, if the tubes are not opened. For the LAMP reaction, these approaches are not acceptable, and therefore we named such a reaction pseudo-hemi-nested LAMP (phn-LAMP). Its advantage over MIP-LAMP variants is that only two annealing sites are added (Figure 8); this approach does not significantly increase the length of the target and makes it comparable to LAMP variants involving loop or stem primers but at the same time provides a much higher level of DNA amplification, as readers can see from the following modeling of this reaction.

In this case, the character of DNA amplification during the LAMP reaction will be the same both for primers FIP and eFIP (extra) together with a single BIP and vice versa: with a single FIP together with BIP and eBIP.

**Figure 9:**
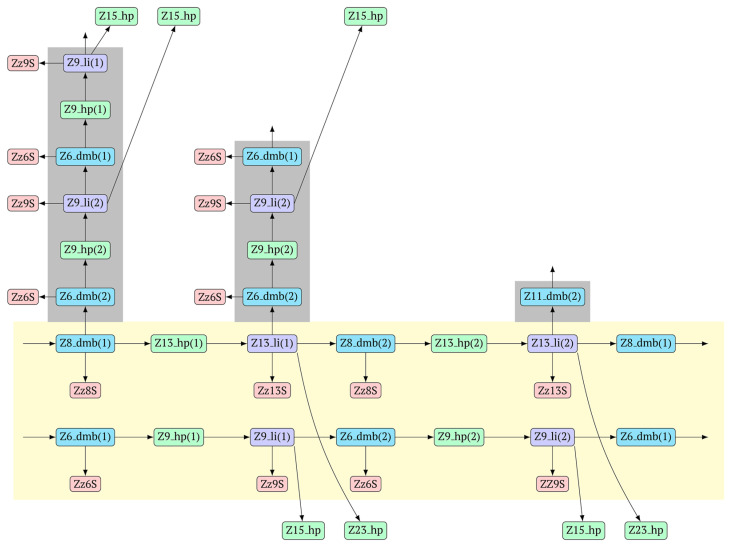
The cascade of cyclic reactions occurring in phn-LAMP. The yellow background shows the main sequence of LAMP cycle events, starting with the entry into the cyclic process of structures **Z8_dmb(1)** and **Z6_dmb(1)** (almost simultaneously), which formed during preamplification. The gray background, on the basis of the number of transformations with time, highlights the later-beginning amplification processes proceeding within the main cycle until the full cycle of the main reaction is completed. Terminated amplicons are red. Horizontal lines delimit the main reaction cycling cascade, beyond which lamplicons Z15_hp and Z23_hp enter the outer contour as they proceed to the NGEL stage (other explanations are in the text).

Nevertheless, for a better explanation of this type of LAMP reaction, it is also necessary to switch to the previously used coding of zones and primers. Thus, in phn-LAMP with the single-letter coding in one of the possible types of FIP and BIP primers’ arrangement, the target would look like this: 5′-…FABCDMNE…-3′, while the primers would be F, bA, Cd, Mn, and e. The initial steps of the preamplification phase can be displayed as follows: 
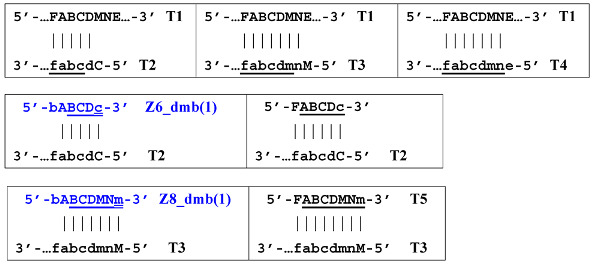


As a result, two types of dumbbell-like structures, **bABCD****c** [Z6_dmb(1)] and **bABCDMN****m** [Z8_dmb(1)], will be generated during the preamplification phase of the phn-LAMP reaction. When they enter the LAMP cycle through a series of processes, structures symmetric to them—**Cdcba****B** [Z6_dmb(2)] and **Mnmdcba****B** [Z8_dmb(2)]—will appear. The increment of lamplicons during one main LAMP cycle in phn-LAMP is illustrated in Figure 9.

**Figure 10:**
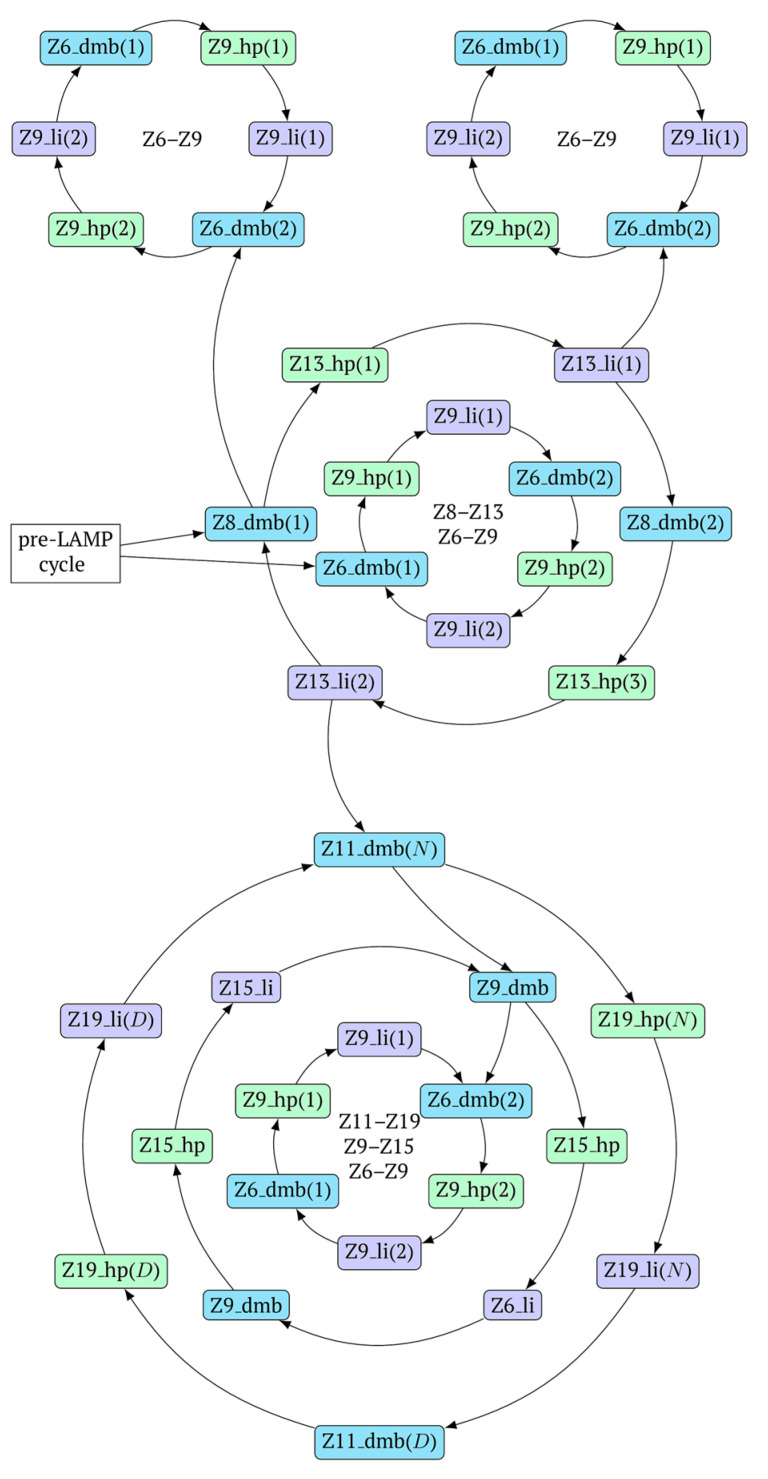
A simplified scheme of LAMP cycle progression in the phn-LAMP reaction with arising subcycles.

In fact, the transition of the phn-LAMP reaction to the exponential phase results in two LAMP cycles in one, accompanied by subcycles, as depicted in the simplified scheme of these processes in Figure 10. The Z11_dmb(2) lamplicon, which generates its own subcycles, deserves special attention.

The phn-LAMP subtype designed for detection of eight zones in a target proceeds in a way similar to the classic LAMP reaction, with a difference being that after preamplification, dmb structures of two sizes—**Z8_dmb(1)** and as its derivative **Z6_dmb(1)**—will initially emerge in the reaction mixture. The **Z6_dmb(1)** structure behaves similarly to that in the classic LAMP reaction, whereas **Z8_dmb(1)** will immediately generate another **Z6_dmb(2)** structure, which will come again to **dmb(2)** via **dmb(1)**.**Z8_dmb** will then engender three more Z6_dmb-like structures.

In the phn-LAMP reaction, taking into account some presumed delay of strand elongation (which is highlighted with the gray background), 18 terminated lamplicons are formed (Zz6S: eight, Zz8S: two, Zz9S: five, and Zz13S: two) and 7 other lamplicons (Z15_hp: five and Z23_hp: two) go out into the NGEL phase. In contrast, in the classic LAMP reaction with a pair of inner primers (FIP and BIP), the number of lamplicons is much smaller: Zz6S, Zz9S, and Z15_hp, two of each. The number of larger lamplicons entering the next loop is also important for subsequent increased multiplication of strands (in the LAMP reaction), which further enhances the exponential nature of such amplification. As for the main type of **Z6_dmb** lamplicon, in the classic LAMP reaction, the number of copies of such lamplicons that entered each LAMP loop is the same as the number of such lamplicons that exit. There is the same situation with **Z8_dmb** lamplicons in phn-LAMP, but in this reaction, instead of one **Z6_dmb** lamplicon that entered, five times more of them come out of each LAMP cycle. In addition to them, another new structure, **Z11_dmb**, emerges in the next cycle.

With the help of the single-letter coding, it is convenient to trace the development of phn-LAMP in a LAMP cycle. Because the **bABCDc** lamplicon [Z6_dmb(1)] that formed during the preamplification phase of phn-LAMP behaves similarly to that in the normal LAMP reaction, its transformations will not be discussed here. As for the **bABCDMNm** [Z8_dmb(1)] lamplicon, Z6_dmb lamplicons periodically arise from it and will also repeat the standard LAMP cycle in Figure 9; they are highlighted by means of the gray background of different heights in accordance with the processes occurring in the main LAMP subcycle.

Thus, the following structures emerge from Z8_dmb(1): 
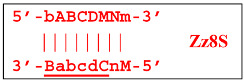


When the Mn primer is annealed, a terminated Zz8S lamplicon is produced, and when the Cd primer is annealed before it, a displaced Z6_dmb(2) strand is formed that can initiate a new LAMP cycle: 
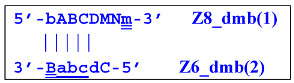


At the same time, the bA primer can be annealed on the Z6_dmb(2) strand, leading to a terminated Zz6S lamplicon: 
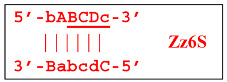


Additionally, for the Z8_dmb(1) structure, there is a probability of self-priming, giving lamplicon Z13_hp(1): 
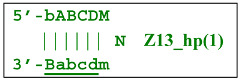
 on the hairpin of which primer Mn is able to anneal (initially with one n zone), leading to the opening of the hairpin and emergence of the Z13_li structure. It also contains the Z8_dmb(2) strand, which can be displaced by the elongation strand from primer bA annealed upstream or by self-priming, leading to the NGEL stage and to lamplicon Z23_hp: 
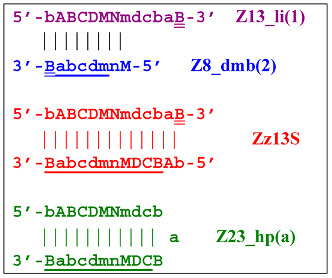


Once the Z8_dmb(2) structure is displaced, it can engender a terminated lamplicon: 
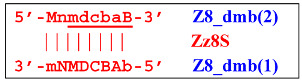
 where Z8_dmb(1) and Z8_dmb(2) are dual mirror twins with respect to each other.

The Z8_dmb(2) strand can also form a new hairpin structure, Z13_hp(2): 
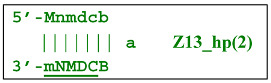


Moreover, the Z13_hp(2) structure—with a loop in the form of zone “a” owing to primer bA annealing on it (first via one zone A)—will cause the opening of the hairpin structure of lamplicon Z13_li(2) and then to terminated lamplicon Zz13S: 
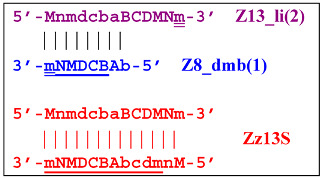


A peculiarity of the Z13_li(2) strand, in contrast to its counterpart Z13_li(1), is that the Cd primer is able to anneal on it, and this event will ensure the formation of a new size range of the lamplicon lineage starting from the Z11_dmb(N) structure, where the type of loop is indicated by a letter instead of numbering “1” or “2”: 
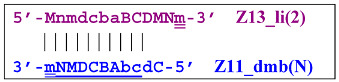


Moreover, within the same LAMP cycle, a new displaceable dumbbell-like Z11_dmb(1) strand will emerge, which will cause the following: from the first LAMP cycle, in the phn-LAMP reaction, an additional somewhat elongated dmb structure will emerge, which already in the next such cycle will turn into Z19_hp, entering the next NGEL step. Through temporary structure Z9_dmb, this molecule will give rise to another basic lamplicon, Z6_dmb(1), initiating a new LAMP cycle.

Regarding strand Z13_li(2), its self-priming will produce hairpin structure Z23_hp, which will exit to the next NGEL contour: 
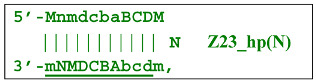
 and derivatives of Z11_dmb will continue transformations already in the next LAMP cycle: 
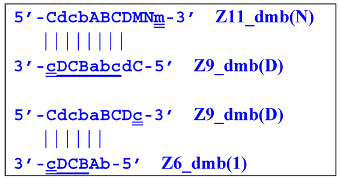


Aside from the formation of the Z6_dmb(1) lamplicon, the Z9_dmb(D) lamplicon will turn into the Z15_hp(D) hairpin structure by annealing on its primer loop. Then, after annealing of the Cd primer (initially with one zone d), it will become the Z15_li(D) structure containing Z9_dmb(D), unable to become Z6_dmb: 
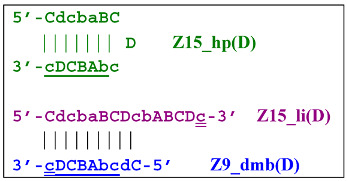


When the LAMP subcycle with Z11_dmb(N) is initiated after annealing and elongation of the Mn primer, a terminated Zz11S lamplicon will emerge: 
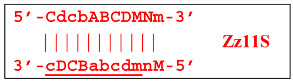
 whereas self-framing will result in a Z19_hp hairpin structure:

It is then linearized, thereby yielding the following structures after annealing and elongation of primers Mn and bA: 
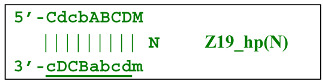


After annealing of the Cd primer, a terminated Zz19S lamplicon will appear: 
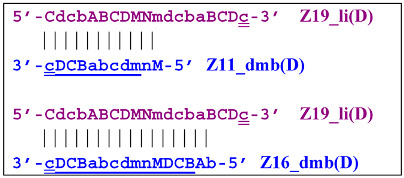


As for the Z16_dmb(D) lamplicon, it will undergo the following transformations—by annealing with three primers (Cd, bA, and Cd) on it—as well as self-priming: 
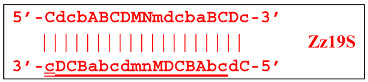


The increase in the size of the lamplicons in the phn-LAMP reaction follows the same logic. When the Z_dmb structure is transformed into its derivative structure Z_hp, the increment takes place by actually doubling the zones minus three zones. That is, starting from Z8_dmb, sizes of subsequent lamplicons in terms of the number of zones will be as follows: Z8 → Z13 → Z23 → Z43 → Z83 → Z163 and so forth. This is the main size series of lamplicons, but as already mentioned above, subsequently, there will be others, starting with lamplicons with an initially larger number of zones.

In this case, the use of one additional inner primer gives a certain advantage, because on the target, there will be a choice of annealing sites or two FIPs and one BIP, or vice versa. It depends on specific sequences of the target. We have previously created primer design software LAMPrimers_iQ (https://github.com/Restily/LAMPrimers-iQ) [23], into which we just added a special option for the selection of primers for phn-LAMP; this feature is designed to select the optimal locations of zones for the three inner primers for this or that type of reaction, as shown in Figure 8. For the selection of annealing sites and for the design of the corresponding primers, it is easier to choose sites for either two FIPs (FIP and eFIP) or two BIPs (BIP and eBIP) than for two loop or stem primers. This is because they must occupy specific locations in the target, thereby narrowing the freedom of choice.

## 3. Conclusions

LAMP is widely used for molecular diagnostics of various etiologic agents, including those of viral nature. During the recent pandemic of infection by the SARS-CoV-2 coronavirus, in addition to RT-PCR, RT-LAMP was widely applied to detect the causative agent. One of the reasons is the isothermal nature of this reaction, which allows POC analysis to be performed without expensive equipment. At the same time, the LAMP reaction is considered as sensitive as or even superior to not only conventional PCR but also its more productive type, called nested PCR. In terms of specificity, LAMP formally outperforms PCR in this parameter by using a larger number of primer zones, provided, of course, that nonspecific amplification is excluded.

LAMP is also used in basic research. For example, it is utilized for genotyping when a single-nucleotide polymorphism (SNP) is detected by means of placement of a discriminating nucleotide at the 5′ end of FIP or BIP primers [24,25,26,27]. An ability of LAMP to detect methylated DNA regions after treatment with sodium bisulfite has been demonstrated [27,28].

Regarding the new reaction type, phn-LAMP, theoretically, it should give faster amplification results (in comparison with the classic LAMP reaction and its variants involving loop and stem primers), revealing the presence of desired targets in an analyzed sample. This property may even expand the scope of practical applications of LAMP. In addition, phn-LAMP requires the same number of annealing zones of primer structures as does LAMP involving loop or stem primers, but the selection of primer zones in a target for the latter techniques is more complicated because the choice of sites is more limited for them. The reason is that this choice is strongly tied to the target sites between F1 and F2, B1 and B2, or FIP and BIP, respectively. With that said, phn-LAMP has a distinct advantage over MIP-LAMP subtypes of this reaction involving multiple primers because phn-LAMP can make do with smaller target sizes and offers a potentially lower likelihood of primer dimer formation.

For even wider use of the LAMP reaction, it is necessary to know all its features, including all possible types of lamplicons, even if they form only theoretically. Moreover, because of differences in analyzed nucleotide sequences among different targets, certain types of lamplicons may be predominant in different LAMP reactions. At the same time, there is no doubt that the theoretical elucidation of LAMP will contribute to the optimization of experiments, thus reducing the time needed for the selection of the necessary parameters. However, it should be taken into account that a non-specific reaction effectively proceeds during isothermal amplification due to the ability of strand-displacement polymerases to multimerize DNA [29,30], and high-quality primer design is required.

## Data Availability

The original contributions presented in this study are included in the article, and further inquiries can be directed to the corresponding authors.

## Abbreviations

The following abbreviations are used in this manuscript:

LAMP	Loop-mediated isothermal AMPlification
PCR	Polymerase chain reaction
SARS-CoV-2	Three-letter acronym
FIP	Forward inner primer
BIP	Backward inner primer
MIP-LAMP	Multiple-inner-primers LAMP reaction
phn-LAMP	Pseudo-hemi-nested LAMP reaction

## References

[1] Notomi T., Okayama H., Masubuchi H., Yonekawa T., Watanabe K., Amino N., Hase T. (2000). Loop-mediated isothermal amplification of DNA. Nucleic Acids Res..

[2] Li M., Ge H., Sun Z., Fu J., Cao L., Feng X., Meng G., Peng Y., Liu Y., Zhao C. (2022). A loop-mediated isothermal amplification-enabled analytical assay for the detection of SARS-CoV-2: A review. Front. Cell. Infect. Microbiol..

[3] Li Y.P., Cao X.J., Luo X., Xie T.A., Liu W.J., Xie S.M., Lin M., Guo X.G. (2023). Evaluation of RT-LAMP Assay for Rapid Detection of SARS-CoV-2. Lab. Med..

[4] Wang L., Tang R., Wang W., Bu L., Sun J., Fu Y., Li M., Yi Z. (2025). Recent developments in isothermal amplification technology for rapid detection of SARS-CoV-2. Anal. Methods.

[5] Nagamine K., Hase T., Notomi T. (2002). Accelerated reaction by loop-mediated isothermal amplification using loop primers. Mol. Cell. Probes.

[6] Gandelman O., Jackson R., Kiddle G., Tisi L. (2011). Loop-mediated amplification accelerated by stem primers. Int. J. Mol. Sci..

[7] Wang D.G., Brewster J.D., Paul M., Tomasula P.M. (2015). Two methods for increased specificity and sensitivity in loop-mediated isothermal amplification. Molecules.

[8] Yang K.L.A., Wu S.Y., Kwok H.C., Ho H.P., Kong S.K. Using Loop-mediated Isothermal DNA Amplification (LAMP) and Spectral Surface Plasmon Resonance (SPR) to Detect Methicillin-resistance *S. aureus* (MRSA). Proceedings of the 2012 International Conference on Biomedical Engineering and Biotechnology.

[9] Yang A.K., Lu H., Wu S.Y., Kwok H.C., Ho H.P., Yu S., Cheung A.K., Kong S.K. (2013). Detection of Panton-Valentine Leukocidin DNA from methicillin-resistant *Staphylococcus aureus* by resistive pulse sensing and loop-mediated isothermal amplification with gold nanoparticles. Anal. Chim. Acta.

[10] Ahsan A., Usman M., Ullah I., Zahur A.B., Malik A.R. (2017). Molecular diagnostic assays for the detection of peste des petits ruminants virus: A concise review. Vet. Sci. Res. Rev..

[11] Suprun E.V., Ptitsyn K.G., Khmeleva S.A., Kurbatov L.K., Shershov V.E., Kuznetsova V.E., Chudinov A.V., Radko S.P., Lisitsa A.V. (2025). Loop-mediated isothermal amplification with tyrosine modified 2′-deoxyuridine-5′-triphosphate: Detection of potato pathogen *Clavibacter sepedonicus* by direct voltammetry of LAMPlicons. Microchem. J..

[12] Notomi T., Hase T. (2002). Process for Synthesizing Nucleic Acid. USA Patent.

[13] Becherer L., Borst N., Bakheit M., Frischmann S., Zengerle R., von Stetten F. (2020). Loop-mediated isothermal amplification (LAMP)—Review and classification of methods for sequence-specific detection. Anal. Methods.

[14] Garafutdinov R.R., Chemeris D.A., Mavzyutov A.R., Akhmetzyanova L.U., Davletkulov T.M., Gubaydullin I.M., Chemeris A.V. (2021). LAMP amplification of nucleic acids. I. Two decades of development and improvement. Biomics.

[15] Subramanian S., Gomez R.D. (2014). An empirical approach for quantifying loop-mediated isothermal amplification (LAMP) using *Escherichia coli* as a model system. PLoS ONE.

[16] Gordon M.I., Klemer D.P., Fuller S.L., Chang J.H., Klemer D.R., Putnam M.L. (2019). Mathematical modeling of a real-time isothermal amplification assay for *Erwinia amylovora*. Eng. Rep..

[17] Kaur N., Thota N., Toley B.J. (2020). A stoichiometric and pseudo kinetic model of loop mediated isothermal amplification. Comput. Struct. Biotechnol. J..

[18] Savonnet M., Aubret M., Laurent P., Roupioz Y., Cubizolles M., Buhot A. (2022). Kinetics of Isothermal Dumbbell Exponential Amplification: Effects of Mix Composition on LAMP and Its Derivatives. Biosensors.

[19] Dangerfield T.L., Paik I., Bhadra S., Johnson K.A., Ellington A.D. (2023). Kinetics of elementary steps in loop-mediated isothermal amplification (LAMP) show that strand invasion during initiation is rate-limiting. Nucleic Acids Res..

[20] Wang D. (2016). Effect of internal primer–template mismatches on loop-mediated isothermal amplification. Biotechnol. Biotechnol. Equip..

[21] Lamalee A., Changsen C., Jaroenram W., Buates S. (2023). Enhancement of loop mediated isothermal amplification’s sensitivity and speed by multiple inner primers for more efficient identification of Vibrio parahaemolyticus. MethodsX.

[22] He X., Su F., Chen Y., Li Z. (2022). Novel reverse transcription-multiple inner primer loop-mediated isothermal amplification (RT-MIPLAMP) for visual and sensitive detection of SARS-CoV-2. Anal. Methods.

[23] Akhmetzyanova L.U., Davletkulov T.M., Garafutdinov R.R., Chemeris A.V., Gubaidullin I.M. LAMPrimers iQ (Loop-Mediated Isothermal Amplification Primers iQ). Certificate of State Registration of Computer Program No. 2022617417.

[24] Kuzuhara Y., Yonekawa T., Iwasaki M., Kadota T., Kanda H., Horigome T., Notomi T. (2005). Homogeneous assays for single-nucleotide polymorphism genotyping: Exo-proofreading assay based on loop-mediated isothermal amplification. Yokohama Med. J..

[25] Gill P., Hadian A.A. (2020). AS-LAMP: A New and Alternative Method for Genotyping. Avicenna J. Med. Biotechnol..

[26] Hao L., Shi X., Wen S., Yang C., Chen Y., Yue S., Chen J., Luo K., Liu B., Sun Y. (2025). Single nucleotide polymorphism-based visual identification of *Rhodiola crenulata* using the loop-mediated isothermal amplification technique. Front. Plant Sci..

[27] Zerilli F., Bonanno C., Shehi E., Amicarelli G., Adlerstein D., Makrigiorgos G.M. (2010). Methylation-specific loop-mediated isothermal amplification for detecting hypermethylated DNA in simplex and multiplex formats. Clin. Chem..

[28] Shen X., Xu M., Wang H., Wang H., Shen M., Talap J., Hu H., Zeng S., Gao S., Cai S. (2023). Site-specific detection of circulating tumor DNA methylation in biological samples utilizing phosphorothioated primer-based loop-mediated isothermal amplification. Biosens. Bioelectron..

[29] Garafutdinov R.R., Gilvanov A.R., Sakhabutdinova A.R. (2020). The Influence of Reaction Conditions on DNA Multimerization During Isothermal Amplification with Bst exo- DNA Polymerase. Appl. Biochem. Biotechnol..

[30] Sakhabutdinova A.R., Garafutdinov R.R. (2025). Mechanism of DNA multimerization caused by strand-displacement DNA polymerases. Anal. Biochem..

